# Development and early feasibility testing of machine-learning algorithms to non-invasively assess hemoglobin levels

**DOI:** 10.1038/s44385-026-00087-5

**Published:** 2026-06-02

**Authors:** Simon Hefner, Philipp Stoffers, Benedikt Langenberger, Alix Holstein, Miha Garafolj, Christina Klötzer, Carmen Herling, Vladan Vucinic, Reinhard Henschler, Ariel Dora Stern, Uwe Platzbecker, Anne Sophie Platzbecker

**Affiliations:** 1 Department of Hematology, Hemostaseology, Cellular Therapy and Infectious Diseases, Leipzig University Hospital, Faculty of Medicine, Leipzig, Germany; 2 University Hospital of Psychiatry, Psychotherapy, and Psychosomatics, Paracelsus Medical University, Salzburg, Austria; 3 Else Kroener Fresenius Center for Digital Health, Faculty of Medicine and University Hospital Carl Gustav Carus, TUD Dresden University of Technology, Dresden, Germany; 4 Digital Health Cluster, Department for Digital Health, Economics & Policy, Hasso-Plattner Institute, University of Potsdam, Potsdam, Germany; 5 Charité - Universitätsmedizin Berlin, Department of Hepatology and Gastroenterology, Campus Virchow-Klinikum (CVK) and Campus Charité Mitte (CCM), Berlin, Germany; 6 Merantix Momentum GmbH, Berlin, Germany; 7 CCCG—Comprehensive Cancer Center Central Germany, Leipzig, Germany; 8 Institute of Transfusion Medicine, University Hospital Leipzig, Faculty of Medicine, Leipzig, Germany; 9 Windreich Department of Artificial Intelligence & Human Health, Icahn School of Medicine at Mount Sinai, New York, NY, USA; 10 Executive Board Office, University Hospital Carl Gustav Carus, TU Dresden, Dresden, Germany; 11 Medizinische Universität Lausitz—Carl Thiem, Cottbus, Germany

**Keywords:** Cancer, Computational biology and bioinformatics, Diseases, Health care, Medical research, Oncology

## Abstract

The HeMonitor study evaluated the feasibility and accuracy of non-invasive hemoglobin (Hb) assessment using image-based techniques and machine learning in patients with hematologic malignancies. A total of 367 patients with hematologic malignancies and 184 healśśthy donors were enrolled, with fingernail and eyelid photographs collected and analyzed using Light Gradient-Boosting Machine (LightGBM) regression models. The best-performing model achieved a residual standard deviation of ±1.02 mmol/L for Hb prediction. Our framework further explored a two-stage concept combining (i) a non-invasive image-based Hb predictor and (ii) a post hoc, rule-basśed corridor aggregation layer integrating EORTC Global Health and Fatigue categories. This exploratory layer was designed to contextualize Hb estimates with patient-reported symptom burden and well-being. Visual analyses suggested that lower Hb levels were generally associated with impaired quality-of-life measures, consistent with the known clinical burden of anemia. Within the QoL subset, the integrated framework showed encouraging concordance with clinician assessments, particularly in borderline Hb ranges. These findings support the feasibility of combining digital biomarkers with patient-reported outcomes for future patient-centered home monitoring strategies, while prospective validation remains necessary.

## Introduction

Routine hemoglobin (Hb) assessment in patients with hematologic malignancies typically requires frequent and invasive blood draws. For patients with myelodysplastic neoplasms (MDS), acute myeloid leukemia (AML), and individuals recovering from chemotherapy, high-frequency complete blood count (CBC) testing (1–2 times weekly) is often required to monitor disease trajectory, detect worsening anemia, and assess the ongoing need for red blood cell (RBC) transfusions. Currently, the lack of reliable, non-invasive home-based options for Hb assessment necessitates regular hospital visits. This requirement can substantially impair patients’ quality of life (QoL), as frequent travel, waiting times, and repeated venipuncture add to the burden of managing both the underlying disease and treatment-related side effects. In recent years, there has been considerable progress in non-invasive Hb measurement techniques, driven by the need for more accessible and patient-friendly approaches. Several groups have explored smartphone-based and computer vision methods for Hb estimation. Wang et al. introduced HemaApp^1^, a smartphone application designed to non-invasively estimate Hb levels using the device camera and multiple light sources to analyze blood color in the user’s finger. In a study involving 31 participants, HemaApp achieved a rank-order correlation of 0.82 with gold-standard blood testing and demonstrated a sensitivity of 85.7% and a precision of 76.5% for anemia screening^1^. These performance metrics are comparable to those of FDA-approved stationary non-invasive Hb measurement devices such as the Masimo Pronto^2^. In the SmartHeLP study by Hasan et al., artificial neural networks (ANNs) were used to estimate Hb levels from smartphone-based fingertip imaging^3^. ANNs are nonlinear data-driven machine learning models capable of identifying complex relationships between input and output variables in supervised and unsupervised settings^4^. In that study, 75 participants placed their fingertips on a smartphone camera with the flash activated, generating short video sequences that were analyzed against invasive reference measurements. The application achieved measurements within ±0.5 g/dL of the gold standard in 92% of cases^3^. Similarly, Chen et al. applied ANN-based methods to smartphone images of the eye and reported a mean absolute error of 1.34 g/dL compared with invasive Hb measurement techniques^5^. The HeMonitor study aimed to develop and evaluate a non-invasive approach for estimating Hb levels from photographs of patients’ fingernails and eyelids as a complementary feasibility framework to traditional laboratory-based methods. Our study extends previous work by combining multimodal image acquisition from fingernail and eyelid photographs with a Light Gradient-Boosting Machine (LightGBM) model^6^. The absence of melanocytes in the eyelid and fingernail regions allows Hb-related chromatic variation to be more directly reflected in tissue coloration^7^. Building on this physiological basis, we applied machine learning methods to extract and analyze subtle image features associated with Hb concentration. The primary goal of this study was to develop and assess an algorithm capable of estimating Hb levels non-invasively, as part of the development and early feasibility testing of a potential tool for routine home monitoring in patients requiring frequent Hb surveillance. Importantly, the HeMonitor study also adopted a patient-centered design and incorporated validated patient-reported outcome measures (PROMs), including the EORTC Global Health Status and Fatigue scales^8^, which were explored post hoc to provide additional contextual information alongside non-invasive, image-based Hb estimates. While our regression-based models demonstrated promising accuracy in estimating Hb levels, we further explored whether QoL data could enhance the interpretability of borderline Hb estimates within an exploratory feasibility framework. This approach acknowledges that anemia can substantially impair QoL, manifesting as fatigue, reduced physical functioning, cognitive limitations, and diminished overall well-being. In this cohort, QoL data were available for 49 of 367 patients. The EORTC Global Health and Fatigue scales are extensively validated across international, multicenter hematology and oncology populations and have demonstrated strong reliability, construct validity, and responsiveness to clinically meaningful changes over time^8^. QoL analyses were exploratory and were not pre-specified in the statistical analysis plan. Unlike studies requiring specialized hardware, HeMonitor relies on standard consumer cameras, which may enhance scalability and accessibility. In collaboration with our hematology-oncology experts, we defined three predefined Hb corridors intended to reflect broad patterns observed in current clinical practice and restrictive transfusion strategies. These corridors were designed as descriptive monitoring categories rather than treatment instructions, resulting in a structured green, yellow, and red framework (green corridor: Hb level > 6 mmol/L; yellow corridor: Hb level > 4.5 mmol/L and < 6 mmol/L; red corridor: Hb level < 4.5 mmol/L). In this cohort of 551 participants, predicted Hb values were mapped to these three corridors using fixed cut-offs. In an additional exploratory step, these Hb corridors were combined with QoL categories in a simple post hoc aggregation framework. Within this conceptual model, red corresponded to increased clinical attention, yellow to an intermediate or ambiguous range, and green to relatively stable Hb levels. This aggregation layer was developed for feasibility testing and hypothesis generation only and should not be interpreted as an automated recommendation system or substitute for clinical judgement. Furthermore, future integration with patient blood management initiatives, such as the German Patient Blood Management Network^9^, may help explore whether non-invasive monitoring approaches can support more patient-centered and resource-conscious anemia care.

## Results

In the HeMonitor study, photographs of fingernails and eyelids were collected from a total of 551 participants, including 367 patients with hematologic malignancies and 184 healthy donors. Among the patients, 266 had no anemia (Hb > 6.21 mmol/L) at the time of imaging, while 101 had anemia (Hb < 6.21 mmol/L). All patients were photographed before receiving any RBC transfusions to ensure that the captured data reflected their baseline Hb levels, under standardized lightning conditions in a room with only artificial origins of light. Healthy donors were photographed pre-donation, under variable lighting conditions, as part of the standard blood donation protocol. The imaging process utilized a standardized setup with a high-resolution camera and consistent white-background calibration to minimize external interference. This dataset provided a robust foundation for developing the model and evaluating its accuracy across a clinically diverse cohort. Three different categories of machine learning models were evaluated in the HeMonitor study: regression models (ridge regression and LightGBM)^6,10–12^, classification models^13,14^, and convolutional neural networks (CNNs)^15–18^.

### Regression models

We evaluated both Ridge regression^11,19^ and LightGBM (LGBM)^6^ models. Ridge regression mitigates multicollinearity and overfitting in linear regression by adding a penalty term to the regression coefficients^12^, whereas LGBM trains an ensemble of decision trees by gradient boosting and is known for its efficiency and high performance in handling large datasets, providing different trade-offs between interpretability and predictive power^6^. Models were independently trained on photographs of fingernails and eyelids, as well as on a combined dataset encompassing both types of images. To evaluate the predictive performance, Ridge regression and LGBM models were initially applied to the fingernail photograph dataset (Table 1). The LGBM model demonstrated superior performance compared to Ridge regression in predicting Hb levels from fingernail photographs. The LGBM model exhibited a slightly smaller standard deviation ( ± 1.06 mmol/L) than Ridge regression ( ± 1.12 mmol/L). Moreover, the mean squared error (MSE) for LGBM was lower at 1.12 compared to 1.27 for Ridge regression, reflecting improved predictive performance. The LGBM model also achieved a higher R² value (0.42 vs. 0.34). The continuous Hb predictions from both models were mapped to the predefined clinical corridors (Red: <4.5 mmol/L, Yellow: 4.5–6.0 mmol/L, Green: >6.0 mmol/L). These findings underscore the superior predictive capability of the LGBM model over Ridge regression for Hb estimation using fingernail photographs. For eyelid photographs, the LGBM regression model similarly outperformed Ridge regression (Table 2). The LGBM model demonstrated a lower standard deviation ( ± 1.03 mmol/L) compared to Ridge regression ( ± 1.06 mmol/L). Additionally, LGBM achieved a lower MSE of 1.06 versus Ridge’s 1.13, and a higher R² value (0.48 vs. 0.45).

**Table 1:** Fingernail Regression

Metric	Ridge regression	LGBM regression
Standard deviation (lower is better)	±1.12 mmol/L	±1.06 mmol/L
Mean squared error (lower is better)	1.27	1.12
R2 (higher is better)	0.34	0.415

All metrics are reported as mean values from 10-fold cross-validation. Standard deviations across folds are not reported due to the exploratory nature of this feasibility study.

**Table 2:** Eyelid Regression

Metric	Ridge regression	LGBM regression
Standard deviation (lower is better)	±1.06 mmol/L	±1.03 mmol/L
Mean squared error (lower is better)	1.13	1.06
R2 (higher is better)	0.45	0.48

All metrics are reported as mean values from 10-fold cross-validation. Standard deviations across folds are not reported due to the exploratory nature of this feasibility study.

**Table 3:** Combined Regression

Metric	Ridge regression	LGBM regression
Standard deviation (lower is better)	±1.05 mmol/L	±1.02 mmol/L
Mean squared error (lower is better)	1.10	1.05
R2 (higher is better)	0.42	0.45

All metrics are reported as mean values from 10-fold cross-validation. Standard deviations across folds are not reported due to the exploratory nature of this feasibility study.

**Fig. 1:**
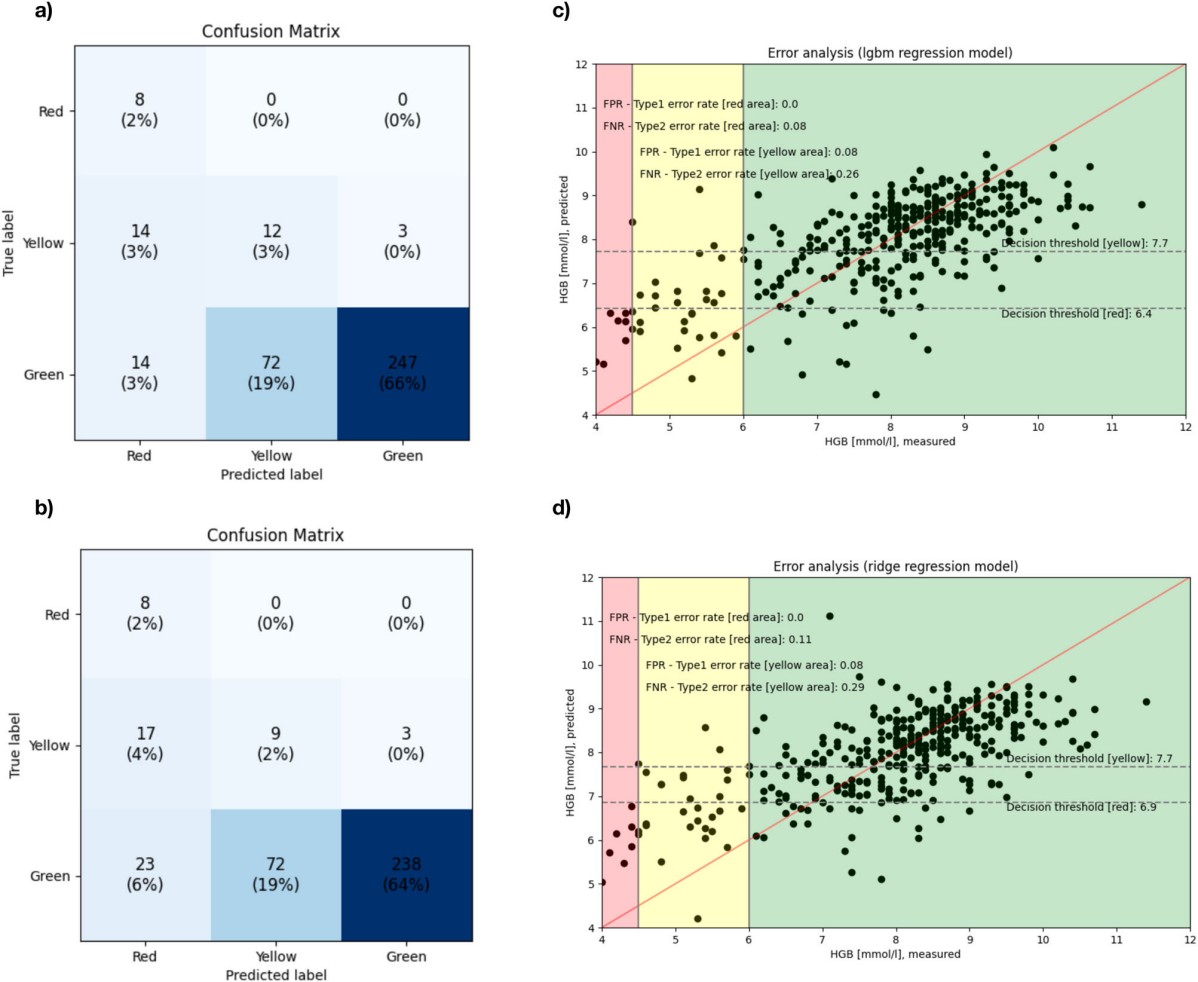
Combined regression model. These matrices highlight the trade-offs in performance between the LGBM and Ridge regression models used in classifying clinical Hb corridors. All metrics are reported as mean values from 10-fold cross-validation. Standard deviations across folds are not reported due to the exploratory nature of this feasibility study. **a** Post-discretized regression to corridor confusion matrix for the combined LightGBM Regression Model; Dataset *n* = 551; 10-fold cross-validation.Continuous Hb predictions from the regression model are mapped to corridor labels using predefined thresholds and thereby illustrate the classification performance of the LightGBM regression model when predicting Hb levels into three clinical corridors: Red (Hb < 4.5 mmol/L), Yellow (Hb 4.5–6.0 mmol/L), and Green (Hb > 6.0 mmol/L). The elements along the diagonal, from the upper left to the lower right, represent correct classifications. A total of 267 instances were correctly classified, with the majority (247) in the Green category. The model achieved a 100% accuracy in the Red corridor, approximately 41.4% in the Yellow corridor, and 74.2% in the Green corridor. The color-coded matrix highlights correct classifications along the diagonal, underscoring the model’s robustness in predicting stable Hb levels (Green) while identifying areas for improvement in borderline cases (Yellow). **b** Confusion Matrix for the combined Ridge Regression Model, similar to (**a**), also same evaluation setup as (**a**): A total of 255 instances were correctly classified, with the majority (238) in the Green category. The model achieved a 100% accuracy in the Red corridor, 31.0% in the Yellow corridor, and 71.5% in the Green corridor. **c** Error Analysis for LightGBM Regression Model; Dataset *n* = 551; 10-fold cross-validation. (Note: The decision thresholds shown in (**c**, **d**) are the predefined clinical corridor boundaries (4.5 and 6.0 mmol/L) used for post-hoc mapping of continuous regression outputs. They were not tuned or optimized for model performance.)The scatterplot compares measured Hb levels (x-axis) to predicted Hb levels (y-axis) using the LightGBM model. Decision thresholds are overlaid (Red: Hb < 4.5 mmol/L, Yellow: Hb 4.5–6.0 mmol/L, Green: Hb > 6.0 mmol/L). Key error metrics, such as Type 1 (false positives) and Type 2 (false negatives) error rates, are annotated for each corridor. The LightGBM model achieves lower Type 2 error rates compared to Ridge regression. The regression line demonstrates the overall fit between predicted and measured Hb levels. **d** Error Analysis for Ridge Regression Model; Dataset *n* = 551; 10-fold cross-validation.: Similar to (**c**), this plot evaluates the Ridge regression model’s predictions. The Ridge model exhibits slightly higher Type 2 error rates (0.11 in red, 0.29 in yellow) compared to LightGBM, indicating more misclassifications, particularly in borderline cases.

When models were trained on a combined dataset of fingernail and eyelid photographs, LGBM once again demonstrated superior performance. It exhibited a lower standard deviation ( ± 1.02 mmol/L) compared to Ridge regression ( ± 1.05 mmol/L) and achieved a lower MSE of 1.05 against Ridge’s 1.10. Moreover, LGBM obtained a higher R² value (0.45 vs. 0.42), indicating better explanatory power (Table 3). Analysis of the confusion matrices for the combined model, computed after discretizing the regression model’s continuous Hb predictions into corridor labels, revealed that LGBM misclassified fewer instances overall (Fig. 1a, b). Specifically, LGBM misclassified 14 Yellow and 14 Green cases as red, compared to Ridge’s 17 Yellow and 23 Green misclassifications. Additionally, LGBM correctly classified greener instances (247 vs. 238 for Ridge). Both models demonstrated identical Type 1 error rates; however, LGBM exhibited lower Type 2 error rates in the red and yellow regions (0.08 and 0.26, respectively) compared to Ridge regression’s 0.11 and 0.29 (Fig. 1c, d).

### Classification model

**Fig. 2:**
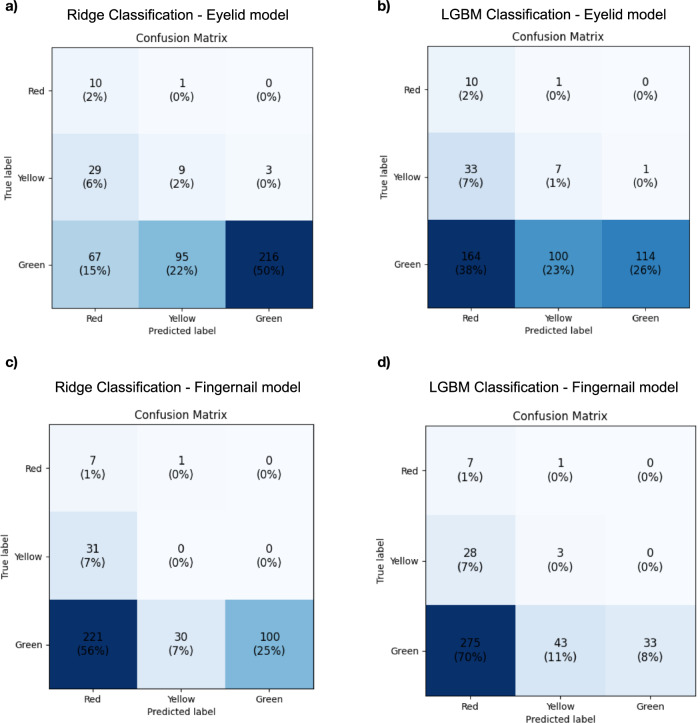
Classification models. Confusion matrices for Ridge and LightGBM classifiers using eyelid and fingernail photos. Dataset n = 551; 10-fold CV; Classes: Red < 4.5; Yellow 4.5–6.0; Green > 6.0 mmol/L. All metrics are reported as mean values from 10-fold cross-validation. Standard deviations across folds are not reported due to the exploratory nature of this feasibility study.(a) Ridge Classification—Eyelid Model, similar to 1 **a**: The confusion matrix highlights the Ridge classification model’s performance using eyelid photographs. A total of 235 instances were correctly classified, with the majority (216) in the Green category. The model achieved approximately 76.9% accuracy in the Red corridor, 18.4% in the Yellow corridor, and 50.1% in the Green corridor. **b** LGBM Classification—Eyelid Model: A total of 131 instances were correctly classified, with the majority (114) in the Green category. The model achieved approximately 67% accuracy in the Red corridor, 14.9% in the Yellow corridor, and 26.3% in the Green corridor. **c** Ridge Classification–Fingernail Model: A total of 107 instances were correctly classified, with the majority (100) in the Green category. The model achieved approximately 87.5% accuracy in the Red corridor, 0% in the Yellow corridor, and 31.3% in the Green corridor. **d** LGBM Classification—Fingernail Model: This model achieved the lowest overall performance, with 43 correctly classified instances. A total of 43 instances were correctly classified, with the majority^33^ in the Green category. The model achieved ~70% accuracy in the Red corridor, 9.7% in the Yellow corridor, and 8% in the Green corridor. Misclassifications are widespread, particularly between Yellow and Green categories.

We further explored the relationship between color categories and anemia conditions in the dataset by employing classification models^13^. These models categorized individuals into one of three predefined anemia categories (Green corridor: Hb level > 6 mmol/L; Yellow corridor: Hb level >4.5 mmol/L and <6 mmol/L; red corridor: Hb level <4.5 mmol/L) using engineered features and metadata. Four classification models were trained^1^: Ridge and^2^ LightGBM models for eyelid photographs, and^3^ Ridge and^4^ LightGBM models for fingernail photographs. The performance of these models is summarized in confusion matrices (Fig. 2). For eyelid-based models, significant differences were observed between LightGBM and Ridge classification. The LightGBM model misclassified 164 Green instances as Red and 33 Yellow instances as Red, while Ridge classification misclassified 67 Green and 29 Yellow instances. Ridge classification outperformed LightGBM in correctly identifying Green instances (216 vs. 114). Both models demonstrated identical Type 1 error rates of 0.0 (Fig. 2a, b). For fingernail-based models, similar patterns emerged. The LightGBM model misclassified 275 Green instances as Red and 43 Green instances as Yellow, whereas Ridge classification misclassified 221 Green instances as Red and 30 as Yellow. Ridge classification also correctly identified more Green instances (100 vs. 33 for LightGBM). Again, both models maintained Type 1 error rates (false positive rate, proportion of cases where the model predicted the Green corridor even though the true Hb corridor was Red) of 0.0 (Fig. 2c, d). Table 4 presents a comparison of F1-scores. For eyelid photographs, the Ridge classification model achieved an F1-score of 0.65, outperforming the LightGBM model, which scored 0.42. Similarly, for fingernail photographs, the Ridge classification model scored 0.40, compared to 0.16 for LightGBM. These results highlight the relative strengths and weaknesses of each classification approach, with Ridge classification demonstrating superior performance in this dataset.

**Table 4:** Classification Results

F1-Score	Ridge Classification	LGBM Classification
Eyelid photographs	0.65	0.42
Fingernail photographs	0.4	0.16

All metrics are reported as mean values from 10-fold cross-validation. Standard deviations across folds are not reported due to the exploratory nature of this feasibility study.

Note: The overall F1-scores are reported. The dataset exhibits class imbalance, with the Green corridor (Hb > 6.0 mmol/L) being the most prevalent. Therefore, these aggregate scores should be interpreted with caution, and class-specific performance is detailed in the confusion matrices (Fig. 2).

### Convolutional neural network (CNN)

In this study, we explored two approaches to CNN implementation. The first involved designing a shallow CNN architecture from scratch, trained on either all three RGB (Red, Green, Blue) channels or a single hue channel. The second approach used a pre-trained feature extraction backbone, where only the final classification layer was trained. However, neither approach achieved convergence on the validation set, with both yielding a mean squared error (MSE) of ~2.9, considerably higher than the baseline performance achieved through an elaborate feature engineering pipeline.

### Post hoc integration of quality-of-life signals for corridor aggregation

**Fig. 3:**
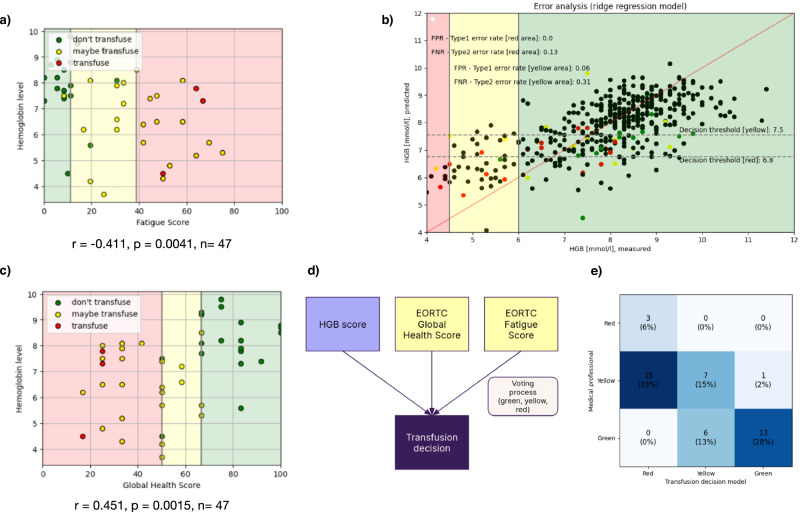
Hybrid corridor-output: post‑hoc integration of EORTC QoL with non‑invasive Hb predictions. All metrics are reported as mean values from 10-fold cross-validation. Standard deviations across folds are not reported due to the exploratory nature of this feasibility study. Note: The scatterplots in (**a**) and (**c**) are presented for illustrative and hypothesis-generating purposes only. Formal correlation statistics were not pre-specified and are not reported. **a** Hb Level vs. Fatigue Score; QoL subset *n* = 47 (pairwise complete). The scatterplot is shown for exploratory purposes; no formal correlation statistics are provided.Scatter plot visualizing the relationship between EORTC Fatigue Scores (x-axis) and Hb levels (y-axis), categorized by transfusion decisions. Data points are color-coded into three categories: “don’t transfuse” (Green), “maybe transfuse” (yellow), and “transfuse” (red). The plot demonstrates how fatigue scores are integrated into transfusion recommendations, with higher fatigue scores generally aligning with lower Hb levels and increased transfusion likelihood. **b** Error Analysis—Ridge Regression Model: Scatter plot comparing measured Hb levels (x-axis) to predicted Hb levels (y-axis) for the Ridge regression model. Data points are color-coded into the same three categories as in (**a**). Decision thresholds (red and yellow regions) are overlaid, with key metrics such as Type 1 and Type 2 error rates annotated for each corridor. In (**b**, **c**) it can be observed that QoL values (e.g., yellow) and predicted Hb values (e.g., Green) do not always align. In cases where the predicted Hb values fall within the yellow range, the additional consideration of QoL values can facilitate or guide the transfusion decision. Therefore, the overlapping representation of the graphs was chosen to visually illustrate these relationships. **c** Hb Level vs. Global Health Score; QoL subset *n* = 47 (pairwise complete). The scatterplot is shown for exploratory purposes; no formal correlation statistics are provided: Scatter plot visualizing the relationship between EORTC Global Health Scores (x-axis) and Hb levels (y-axis), categorized by transfusion decisions, similar to (**a**). Higher Global Health Scores typically align with higher Hb levels, supporting less frequent transfusion recommendations. **d** Post‑hoc rule‑based voting aggregator: Schematic of the post‑hoc decision layer. The LightGBM Hb predictor (trained on image features only) yields a predicted Hb corridor (Green/Yellow/Red). EORTC Global Health and Fatigue scores are independently mapped to corridors via pre‑specified cut‑points. A majority vote across the three agents determines the final recommendation; when there is no majority, a conservative tie‑break selects the more severe class (Red > Yellow > Green). QoL is not an input to the regression model. The three agents are equally weighted, and the system is fully deterministic and rule-based. **e** Confusion Matrix for Transfusion Decision Model vs. Medical Professional Decisions: Correct classifications are observed for 3 Red, 7 Yellow, and 13 Green cases, totaling 23 correctly classified instances. Misclassifications primarily occur in the Yellow category, with 15 cases classified as Red and 1 case classified as Green. Additionally, 6 Green cases are misclassified as Yellow. The model demonstrates strong alignment with medical professionals in the Green category but shows a tendency toward conservative predictions, often recommending transfusions in borderline cases, as reflected by the misclassification of Yellow cases as Red.

To contextualize Hb predictions within patient-experienced disease burden, we performed a post hoc integration of quality-of-life (QoL) measures into corridor interpretation. Exploratory visual analyses were conducted in the subset of patients with complete QoL data (*n* = 47) to assess the relationship between Hb levels and EORTC Global Health and Fatigue scores. QoL variables were not included as predictors in the Hb regression models and were used exclusively for downstream contextualization. Baseline characteristics of this subset were comparable to the overall cohort. For clinical interpretability, EORTC scores were categorized into three intuitive strata. For Global Health, low QoL was defined as 0–45, moderate QoL as 45–65, and high QoL as 66–100. For Fatigue (reverse-scaled), low QoL corresponded to 39–100, moderate QoL to 17–38, and high QoL to 0–16. These categories were not used for model training but were incorporated post hoc into a rule-based aggregation layer (Fig. 3d). Exploratory visual analyses suggested that lower Hb levels were generally accompanied by impaired Global Health and increased Fatigue (Fig. 3a, c), consistent with the known symptomatic burden of anemia. Formal correlation testing was not pre-specified and is therefore not reported. Accordingly, these analyses are intended for hypothesis generation only. Predicted Hb values were translated into three predefined clinical corridors using fixed cut-offs (Fig. 3b). Hb > 6 mmol/L defined the Green corridor, reflecting a restrictive transfusion paradigm in which red blood cell (RBC) transfusion is typically not indicated. Hb 4.5–6 mmol/L defined the Yellow corridor, representing a clinically ambiguous zone in which management decisions frequently require individualized clinical assessment. Hb < 4.5 mmol/L defined the Red corridor, corresponding to levels at which transfusion is commonly considered because of increasing physiological risk. These thresholds reflect current clinical practice and serve as feasibility anchors rather than prescriptive decision rules. To integrate physiological measurement with patient-reported symptom burden, we implemented a post hoc rule-based aggregation combining three components: predicted Hb corridor, Global Health corridor, and Fatigue corridor. Each component contributed equally and was combined by majority vote. In the absence of a majority, a conservative rule selected the more severe corridor (Red > Yellow > Green), reflecting a safety-oriented escalation principle. For example, a Yellow Hb prediction combined with Red QoL strata resulted in a Red aggregated label. Within the QoL subset, aggregated corridor labels were compared with clinician assessments (Red: transfusion indicated; Yellow: transfusion likely; Green: no immediate intervention). The integrated approach demonstrated encouraging concordance with clinician judgement, particularly in borderline Hb ranges, where management decisions are most nuanced (Fig. 3e). These findings suggest that incorporating patient-reported outcomes alongside non-invasive Hb estimation may improve clinical interpretability and better align algorithmic outputs with real-world clinical reasoning.

## Discussion

This study presents a proof-of-concept framework for non-invasive, image-based Hb monitoring intended for future longitudinal home use in patients requiring frequent laboratory surveillance. HeMonitor was not designed to replace laboratory Hb measurements or to automate transfusion decisions. Rather, its purpose is to detect directional Hb trends and early deterioration in a remote setting, thereby supporting clinical reassessment when necessary. The clinical relevance of prediction error must therefore be interpreted within this application context. In transfusion medicine, decisions are rarely determined by absolute Hb thresholds alone; they incorporate symptoms, comorbidities, disease trajectory and patient-reported outcomes. The predefined Hb corridors (Green >6 mmol/L, Yellow 4.5–6 mmol/L, Red <4.5 mmol/L) were intentionally selected from a clinical perspective as conservative guidance bands reflecting restrictive transfusion strategies rather than prescriptive decision cut-offs. Restrictive strategies, typically employing lower transfusion thresholds, have consistently been shown to reduce unnecessary RBC exposure without compromising safety and to minimize complications such as transfusion-associated circulatory overload, infection, immunomodulation and iron overload^20–24^. The corridor framework, therefore, aligns with contemporary patient blood management principles. Within a home-monitoring paradigm, laboratory-grade precision is not the primary objective. Clinically meaningful performance depends on reliable detection of Hb decline and appropriate identification of patients requiring reassessment. An error margin on the order of ±1 mmol/L may be acceptable in this context if it enables early clinical review while avoiding unnecessary hospital visits in patients with stable Hb levels. The system is deliberately conservative: in ambiguous cases, escalation to clinical reassessment is favored over missed anemia progression. This precautionary bias reflects safety-first principles in remote monitoring. The physiological premise underlying HeMonitor, that Hb concentration modulates color intensity in anatomical regions with minimal melanocyte interference, was supported by the observed association between image-derived hue features and laboratory Hb values. Among evaluated modalities, eyelid images consistently provided the strongest predictive signal. Fingernail-based predictions were weaker, even when aggregated across multiple nails. Combining fingernail and eyelid images resulted in only marginal numerical improvement in residual standard deviation (from ±1.03 to ±1.02 mmol/L in the LightGBM model). Given the absence of retained fold-level outputs, these differences must be interpreted descriptively rather than inferentially. From a practical perspective, eyelid-based imaging alone may represent the most efficient and clinically feasible approach, while multimodal acquisition may offer incremental robustness under variable acquisition conditions without substantial performance gain under controlled settings.

From a modelling standpoint, regression approaches consistently outperformed classification models, which is expected given the continuous nature of Hb as a biological variable. Feature-engineered regression models demonstrated superior stability compared with direct multi-class classification. LightGBM achieved modestly better predictive metrics than Ridge regression across datasets, although differences were small. In moderate-sized, low-signal clinical datasets, simpler models offer advantages in interpretability, computational efficiency and reduced risk of overfitting. The marginal performance gain of a more complex ensemble method must therefore be weighed against these considerations. Ridge regression, as a linear model, offers interpretability and stability^12^, while LGBM, a gradient boosting model, effectively captures complex nonlinear relationships within the data^6^. Interpretability could be enhanced using SHAP (Shapley Additive exPlanations) analysis. SHAP is a method to quantify how much each input feature contributes to a model’s predictions^20^. SHAP quantifies each feature’s contribution to individual predictions and makes otherwise “black-box” algorithms more interpretable^21^. This technique is especially useful for explaining complex models (like LightGBM) in a way that humans can interpret, thus offering more transparency in the results^20^. CNNs did not converge reliably. This negative finding is informative rather than incidental. Hb-related color variation constitutes a low signal-to-noise problem, and the available dataset size is insufficient for robust training of high-parameter deep architectures. The superior performance of feature-engineered models likely reflects strong inductive bias introduced through physiologically informed hue representations and dimensionality reduction, which improves data efficiency and mitigates overfitting. In this context, domain-informed feature engineering appears better suited than end-to-end deep learning for capturing subtle chromatic signals associated with Hb concentration. A conceptual innovation of this study is the post-hoc integration of validated QoL measures into corridor interpretation. Importantly, QoL variables were not incorporated into model training but were integrated in a transparent, deterministic aggregation layer. This preserves model interpretability, avoids label leakage and allows objective physiological estimates to be contextualized with patient-reported symptom burden. The exploratory QoL-augmented corridor aggregation demonstrated improved concordance with clinician assessments, particularly within the clinically ambiguous yellow corridor. Transfusion decisions in this range are inherently individualized. A patient with Hb of 5.0 mmol/L and preserved well-being may be safely observed, whereas a similarly measured patient with substantial fatigue or impaired global health may benefit from RBC transfusions. By integrating Hb estimates with validated patient-reported outcomes, the framework reflects real-world hematologic reasoning and moves beyond an exclusively Hb-centric paradigm. Nevertheless, this aggregation layer should be regarded as an exploratory feasibility construct rather than a clinical decision aid, as additional determinants, including comorbidities, anemia etiology and Hb trajectory, were intentionally not modeled. Building on this foundation, recent studies underscore the critical interplay between Hb levels, transfusion strategies, and QoL outcomes in hematological patients. For instance, Buckstein et al.^22^ demonstrated a positive association between higher Hb levels and improved QoL in transfusion-independent MDS patients, while transfusion-dependent patients exhibited more variable QoL improvements. Similarly, Abel et al.^23^ and Haring et al.^24^ highlighted the nonlinear relationship between Hb levels and QoL, suggesting that other factors, such as comorbidities and individual adaptation to anemia, may significantly influence QoL outcomes. Furthermore, Romanenko et al.^25^ observed that, despite achieving threshold Hb levels, symptoms of hypoxia may persist in some patients, emphasizing the necessity of individualized transfusion strategies, a need that aligns closely with the capabilities of HeMonitor.

To situate our findings within the rapidly evolving field of smartphone-based non-invasive Hb estimation, it is important to examine methodological and performance differences across prior approaches. Chen et al.^5^ developed a deep learning–based system combining Efficient Group Enhanced U-Net (EGE-UNet) for eyelid segmentation^26^ with a Delta Hemoglobin AdaIN (DHA)(C3AE) architecture for Hb prediction^27^. Their model achieved a mean absolute error of 1.34 g/dL, a mean squared error of 2.85 g/dL², a root mean square error of 1.69 g/dL and an R² value of 0.34. The segmentation backbone demonstrated strong technical performance, with a mean intersection-over-union of 0.78, an F1 score of 0.87 and an accuracy of 0.97. In contrast, the present study employed physiologically grounded feature engineering combined with Ridge regression^12^ and LightGBM^6^. The LightGBM model trained on combined eyelid and fingernail features achieved a residual standard deviation of ±1.02 mmol/L (~±1.64 g/dL), an MSE of 1.05 mmol²/L² (~2.66 g/dL²) and an R² of 0.45. Although direct statistical comparison is limited by differences in cohorts and evaluation protocols, the proportion of explained variance in our model exceeds that reported by Chen et al., despite the absence of end-to-end deep segmentation and prediction pipelines. SmartHeLP^3^ adopted yet another paradigm, using smartphone video recordings of the fingertip under flash illumination and artificial neural networks trained on RGB pixel intensities. In a cohort of 75 participants, SmartHeLP achieved a rank-order correlation of 0.93 with laboratory Hb measurements and classified 92% of values within ±0.5 g/dL of the reference standard, reporting sensitivity of 94% and specificity of 96% for anemia detection. However, regression metrics such as MSE or R² were not reported, precluding direct quantitative comparison. Furthermore, video-based flash acquisition differs substantially from static photograph–based approaches. In contrast, the present study analyzed 551 individuals, including patients with hematologic malignancies and healthy donors, providing a larger and clinically more heterogeneous dataset. These differences in acquisition modality, cohort composition and reporting standards underscore the need for harmonized benchmarking metrics in this field. HemaApp^1^ utilized smartphone cameras augmented with external hardware, including infrared LEDs and incandescent lighting, and was evaluated in a cohort of 31 participants. While promising, the requirement for hardware augmentation introduces scalability constraints. HeMonitor operates using standard consumer smartphone cameras without dedicated illumination modules, enhancing accessibility and potential deployment in remote or resource-limited settings. The absence of hardware support, however, places greater emphasis on algorithmic robustness under variable real-world conditions, reinforcing the necessity of prospective external validation.

Several limitations warrant consideration. Imaging was performed under standardized lighting and acquisition conditions to ensure internal validity during feasibility testing. Real-world smartphone environments introduce variability in lighting, camera calibration, skin pigmentation and user behavior, all of which may affect generalizability. External validation in diverse populations and uncontrolled acquisition settings is essential. The dataset is imbalanced, with overrepresentation of higher Hb values, reflecting clinical practice in which severely anemic patients are often transfused promptly and thus underrepresented in imaging. Prospective enrichment of lower Hb ranges will be necessary to optimize calibration. Fold-level cross-validation outputs were not stored, precluding retrospective calculation of confidence intervals across folds; future studies will incorporate systematic uncertainty quantification. Segmentation accuracy was not quantitatively benchmarked with pixel-level ground truth, as segmentation served as a pragmatic preprocessing step rather than an inferential objective. Finally, QoL analyses were exploratory and based on a limited subset; adequately powered prospective studies with pre-specified hypotheses will be required to validate these associations.

In its current form, HeMonitor should be regarded as a feasibility framework for non-invasive Hb trend monitoring aligned with restrictive transfusion strategies and patient-centered care. Its potential future clinical value lies in enabling longitudinal monitoring outside the hospital, supporting early detection of clinically meaningful Hb decline and reducing unnecessary visits in stable patients. Prospective real-world validation will determine whether this approach can safely complement laboratory diagnostics and contribute to individualized anemia management. By combining physiologically grounded image analysis with transparent integration of patient-reported outcomes, this work illustrates how digital biomarkers may augment, not replace, clinical judgement in hematology and oncology.

## Methods

### Collection of a training dataset

HeMonitor was an open-label, single-center, observational trial and was approved by the institutional review board of University Hospital Leipzig and conducted according to the Declaration of Helsinki. We captured photographs of fingernails and eyelids from both healthy individuals and patients with hematological disorders during the period from October 2021 to March 2022. We classified the photographs into three groups: subgroup A was the healthy control group, with entries from healthy individuals from the blood donor unit (*N* = 184); subgroup B included entries from patients with a hematological malignancy and anemia (Hb levels<6.21 mmol/l) (*N* = 101); and subgroup C included entries from patients with a hematological malignancy but no anemia (Hb level >6.21 mmol/l) (*N* = 266). The healthy control group (subgroup A) consisted of healthy voluntary blood donors at the University Hospital Leipzig. Their skin photo documentation was taken before blood donation under varying illumination conditions. Pre-donation Hb levels were measured using the stationary quick test device CompoLab TS (Fresenius Kabi), which utilizes a photometric method to assess Hb in capillary blood^28^. Patients with hematologic diseases attended the hematology outpatient clinic of the University Hospital of Leipzig for regular chemotherapy or transfusion visits. For these patients, blood sampling was a planned standard procedure on the same day the photographs were taken. Importantly, the photographs were taken before any RBC transfusion. Subjects could participate in the study multiple times, with the requirement that additional photographs and CBCs were taken on different days compared to the first photographs. Thus, a patient could be assigned to group B on the first entry and to group C on the second entry if their Hb level changed accordingly. Furthermore, in addition to the photos of the fingernail and eyelid and the laboratory-based invasive measured Hb values before the blood transfusion, we also collected clinical data from every patient, including age, gender, and a list of their diagnoses (Table 5).

**Table 5:** Proband Characteristics

Proband Subgroups	
Subgroup A: Healthy control group	*N* = 184
Median Age	39 (Range: 18–78)
Median Hb in mmol/l	8.6 (Range: 6.5–11.4)
Female	89/184 (48%)
Male	95/184 (52%)
Subgroup B: Patients with a hematological malignancy and anemia (Hb < 6.21 mmol/l) *at the time of the photographs* (Subgroup B)	*N* = 101
Median Age	62 (Range: 21–83)
Median Hb in mmol/l	5.2 (Range: 3.4–6.2)
Female	51/101 (50%)
Male	50/101 (50%)
Subgroup C: Patients with a hematological malignancy but without anemia (Hb > 6.21 mmol/l *at the time of the photographs* (Subgroup C)	*N* = 266
Median Age	59 (Range: 21–81)
Median Hb in mmol/l	7.9 (Range: 6.3–11.3)
Female	114/266 (43%)
Male	152/266 (57%)
Diagnosis of Patients in Subgroups B + C	
Acute Myeloid Leukemia (AML)	*N* = 96
After Hematopoietic Stem Cell Transplantation (alloSCT)	*N* = 54
* Indications for Transplantation (including but not limited to)*	
- Acute Myeloid Leukemia	*N* = 16
- Myelofibrosis	*N* = 4
- Myelodysplastic Neoplasms	*N* = 4
- Multiple Myeloma	*N* = 3
Multiple Myeloma (MM)	*N* = 47
lower-risk MDS	*N* = 29
higher-risk MDS	*N* = 20
Acute Lymphoblastic Leukemia (ALL)	*N* = 14

All metrics are reported as mean values from 10-fold cross-validation. Standard deviations across folds are not reported due to the exploratory nature of this feasibility study.

The experimental setup for the study used a Sony ILCE-7 camera with a full-frame sensor designed to capture high-resolution images in RAW format. This capability allowed for extensive post-processing flexibility, which is critical for accurate analysis. The photographic environment was set up with a plain white background, specifically selected for white calibration to ensure color accuracy in the images. This setup minimized background interference and enhanced focus on the specific subjects of the photographs.

### Discretization of the target variable

The aim was to develop and evaluate an algorithmic model capable of estimating Hb levels non-invasively from photographic data, as an early feasibility framework for home monitoring that may allow longitudinal comparison with prior measurements and detection of downward Hb trends. While the target variable (Hb) is naturally reported as a continuous variable in mmol/L units, we discretized it into three clinically-relevant categories with the goal of developing a “corridor” model that incorporates not only Hb measures but also QoL and provides patients with action instructions based on their results. We defined the corridors based on clinical experience and according to existing transfusion guidelines as follows: green corridor was defined as a Hb level > 6 mmol/L, indicating no need for immediate medical attention; Yellow corridor was defined as a Hb level >4.5 mmol/L and <6 mmol/L, indicating a doctor’s visit with a likely RBC transfusion; and the red corridor was defined as a Hb level <4.5 mmol/L, indicating the need for a doctor’s visit and a high likelihood for RBC transfusion. This discretization enabled a specialized assessment of individual categories during the model evaluation step, facilitating the computation of relevant metrics. These corridor boundaries were fixed and used exclusively for the post-hoc mapping of continuous Hb predictions to the three clinical categories. They were not tuned or optimized during model development.

### Image cluster analysis

To achieve separation of images of fingernails and eyelids within the shared dataset, we employed a combination of tools, namely torchvision/resnet18, a pre-trained image embedding model^29^; umap, a dimension reduction technique^30^; and k-means, a clustering algorithm^31^. We employed a face landmark model^32^ to extract the eye area from facial images. Finally, we employed clustering analysis again to isolate the exposed eyelid only. This analysis divided our dataset with the extracted eyes into those containing the relevant eyelid region and those that were not useful.

### Feature engineering

**Fig. 4:**
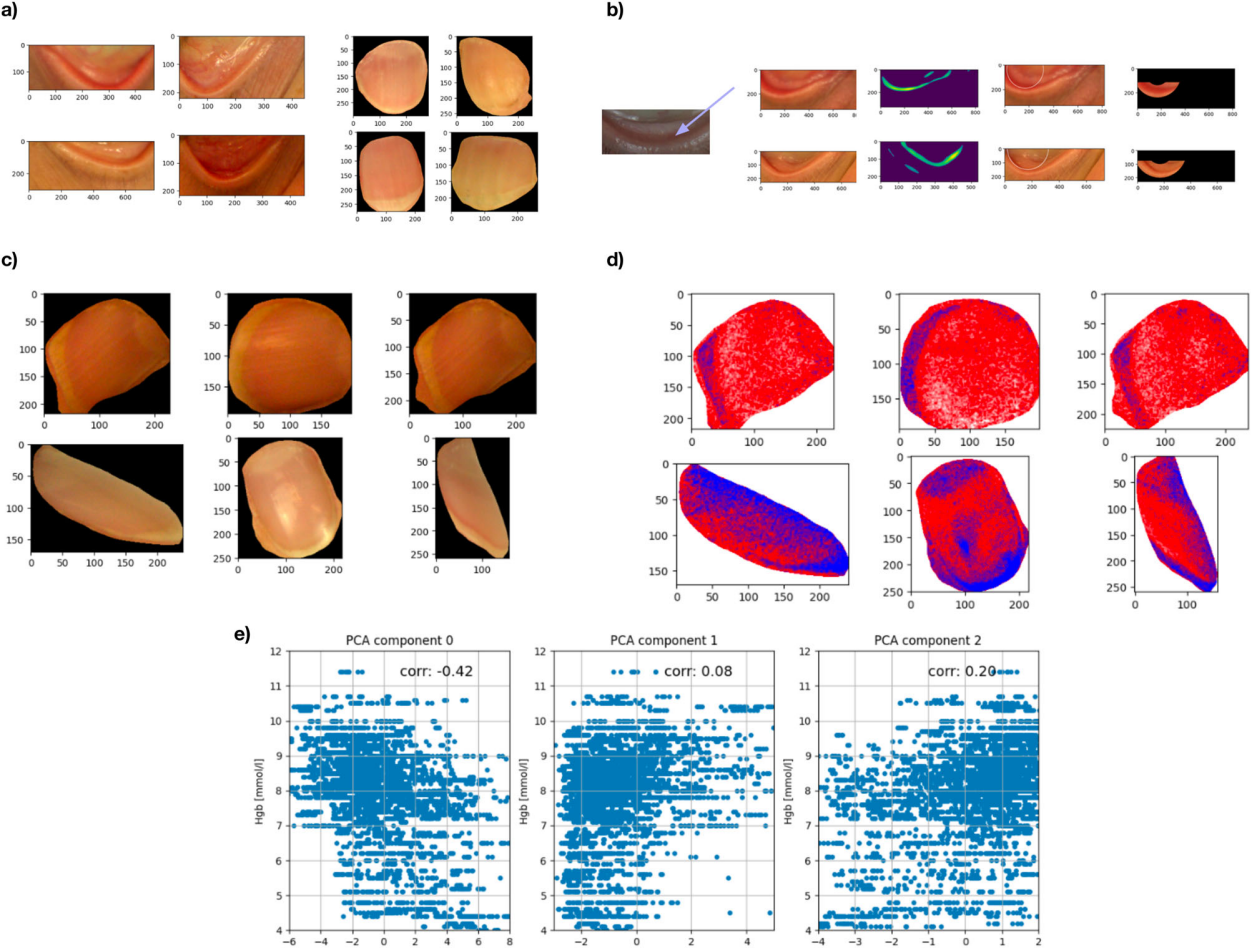
Image engineering. These panels demonstrate the image processing pipeline, from raw data to feature extraction, and the role of PCA in capturing color-based features relevant to Hb level estimation. Workflow: **a** raw eyelid and fingernail photos → **b**, **c** ROI segmentation of palpebral conjunctiva and nail plate with basic QC → **d** HSV hue feature extraction within the ROIs → **e** PCA of the resulting feature space. (Hb regression and QoL voting occur downstream and are not part of this figure). **a** Raw Images of Eyelids and Fingernails: Example images of eyelids and fingernails captured in the study, illustrating the raw input data used for Hb prediction. These images highlight the variability in anatomical features and lighting conditions. **b** Segmentation of Eyelid Regions: The process of isolating relevant eyelid regions from facial images is depicted. The steps include initial raw image processing, edge detection, and the final segmented region used for analysis, ensuring accurate focus on the target area for Hb estimation. **c** Segmented Fingernail Images: Fingernail regions extracted from raw hand images using segmentation techniques. These isolated regions remove irrelevant background features and emphasize the fingernail area critical for Hb prediction. **d** Hue Extraction and Color Mapping: Segmented regions are processed to extract hue features (representing red-to-blue color ratios). The mapped hues are visualized in red and blue to highlight color patterns that correlate with Hb levels, enabling robust feature extraction for modeling. **e** Principal Component Analysis (PCA) on Hue Features: Scatter plots of the first three PCA components derived from hue features, plotted against measured Hb levels. Correlation values for each PCA component are displayed. PCA was used for feature compression and denoising; component-wise explained variance ratios are not reported as they were not retained during this feasibility study. The visualization is illustrative and not intended for statistical inference. **b**, **c** ROI segmentation of the palpebral conjunctiva and nail plate was performed using unsupervised methods (Grounded-SAM, Hough transform, erosion) to isolate regions for hue extraction. Segmentation was used as a preprocessing step to enhance feature quality; no ground-truth annotations were available for quantitative validation (e.g., Dice/IoU).

Feature engineering is a pivotal step in the development of a machine learning model. It entails extracting pertinent information from raw data to create informative features for subsequent modeling tasks. We implemented a feature engineering pipeline designed to capture the essential characteristics of the input images, which were crucial for the regression model. Due to the insufficient useful signal in the original images, direct utilization is not feasible. Therefore, we initially segmented the relevant areas by the mentioned open-source algorithms. We used the Grounded-Segment-Anything algorithm^33^ for fingernail segmentation and MediaPipe’s Face Landmarker model for eye area identification and extraction^32^ (Fig. 4a). The segmented fingernails included regions without underlying blood vessels, such as skin edges, uncut fingernail portions, and minor segmentation errors. To address this, we applied an erosion process, iteratively removing pixel values at the mask’s edges. As a result, the final segmentation mask retained 30% of the pixels from the original mask. To isolate only the relevant area of the eyelid with high precision, we employed more advanced techniques; given the consistent half-circular shape of the eyelid, we utilized a combination of an edge detection algorithm^34^ and the Hough Circle Transformation^35^, which identifies the most fitting circular region within the patch. Our selected relevant eyelid area corresponds to the lower segment of this circle (Fig. 4b).

Segmentation was used exclusively as an unsupervised pre-processing step to isolate regions of interest and improve signal-to-noise ratio for downstream color-based feature extraction. The study was intentionally designed to evaluate end-to-end hemoglobin prediction performance, not anatomical segmentation accuracy. Segmentation quality was therefore assessed qualitatively and optimized pragmatically for robustness rather than pixel-level anatomical fidelity. The lack of quantitative segmentation validation (e.g., Dice/IoU metrics) is acknowledged as a methodological limitation to be addressed in future work.

### Hue representation

Following the segmentation of the images, we converted the segmented regions from the red, green, and blue (RGB) color space to a hue representation. Hue representation is a fundamental concept in color science, characterizing colors based on their position on the color wheel, typically measured in degrees. This metric is integral to models such as HSV (hue, saturation, value) and HSL (hue, saturation, lightness), where hue specifies the type of color. Compared to the RGB model, hue represents the pure color of an image, regardless of its brightness or saturation. While RGB values provide color information, HSV, specifically the hue channel, is preferred for analyzing Hb levels because it separates hue from brightness and intensity, making it easier to focus on actual color changes indicative of variations in Hb. Hue directly represents color type, crucial for analyzing the red-to-blue ratio, and is less affected by lighting conditions compared to RGB. This separation provides a more robust and consistent measure, facilitating a precise and intuitive analysis of Hb levels. Other studies^36,37^ also employed RGB and HSL color models in the context of machine learning applications. The hue conversion is particularly significant as the patient’s Hb levels are influenced by the red-to-blue ratio present in the images. A common method for obtaining hue values involves converting RGB values (Fig. 4c) into the HSV color space (Fig. 4d) and discarding saturation and value channels. This conversion allows us to concentrate on the color information within the selected regions, effectively highlighting the red and blue components. Once we obtained the hue representation, we computed histograms from the hue vectors, providing a concise summary of color distribution in the segmented regions. This data quantifies the prevalence of different hues, particularly red and blue, within the areas of interest, offering insights into their color composition.

### Principal component analysis

To further refine features and reduce dimensionality, we applied principal component analysis (PCA) to the computed histograms. PCA is a dimensionality reduction technique that identifies the most significant components, or principal components, of the data^38^. By applying PCA to our histograms, we transformed the data into a lower-dimensional space while retaining as much relevant information as possible, thereby generating robust features for the data samples. These reduced-dimensional representations served as the final set of features for the envisioned regression model (Fig. 4e). Principal component analysis was applied solely as a feature compression and denoising technique, not for inferential interpretation. Component-wise explained variance ratios were not retained during model development and, therefore, cannot be reported retrospectively. The PCA visualizations are illustrative and not intended to support statistical inference. This limitation is now explicitly acknowledged in the revised manuscript.

### Modelling approach

We evaluated three model families: regression models to predict Hb as a continuous outcome, multi-class classification models to directly assign subjects to discrete health states, and convolutional neural networks (CNNs) as an image-based alternative. Regression models learn relationships between predictor variables and a continuous outcome and are commonly used for data-driven prediction and inference^10^. Whereas the regression models were designed to estimate Hb on a continuous scale, the classification models shift the task to assigning subjects to distinct health states^14^. Ridge classification was used as a regularised baseline to mitigate multicollinearity by penalising model coefficients^19^. In contrast, LightGBM classification was included for its computational efficiency and strong performance in classification tasks on tabular data; LightGBM implements gradient-boosted tree ensembles and is well-suited to large datasets^6^. CNNs were explored as an alternative approach that can learn predictive representations directly from raw input images (e.g., eyelid or fingernail photographs), thereby reducing reliance on manual feature engineering^38^.

### Model evaluation and validation framework

We implemented a 10-fold cross-validation scheme, partitioning the dataset into ten random subsets. In each iteration, nine subsets were used for training, and one subset was used for validation and testing. This process was repeated ten times, with each subset serving as the validation set exactly once. Cross-validation allowed us to assess the model’s performance across various data splits, reducing the risk of overfitting and providing a more accurate evaluation of its generalization capabilities. Moreover, it allowed us to analyze the model’s performance over the full dataset. The dataset was split on a patient ID basis, ensuring that an individual patient was part of either the training or validation set at any given time, thereby preventing information overlap and ensuring robust and reliable evaluation results representative of real-world performance. Cross-validation was performed strictly on a patient-ID basis. All visits, images, and derived samples from a given patient were assigned to the same fold to prevent information leakage between training and validation datasets. Some patients contributed multiple visits over time; these repeated observations remained confined to a single fold. Predictions were generated at the individual image level, including eyelid regions and fingernail regions, and subsequently aggregated using the median operator to yield a single patient-level Hb estimate per validation fold. Aggregation was performed exclusively within validation folds to ensure independence from training data and to improve robustness against outlier image predictions. Only validation metrics (i.e., metrics computed on the validation dataset) were reported; each metric is an average over the validation folds. To align with the project’s objectives and dataset characteristics, we adopted an approach of aggregating individual predictions. Instead of producing multiple predictions for each patient ID, due to multiple images taken or multiple fingernails/eyelids present in a single image, we aggregated these using the median operator, resulting in a single prediction per patient. This aggregation enhances robustness against outlier predictions, providing more trustworthy results. To evaluate the progress and contributions of our experiments, we employed both quantitative and qualitative analyses: *Regression metrics:* A) The standard deviation assessed the dispersion of prediction errors in our regression model. It quantifies how far the model’s predictions deviate, on average, from the actual observed Hb levels. A lower standard deviation implies higher precision, whereas a higher standard deviation suggests greater variability in prediction errors, affecting overall model accuracy. Monitoring the residual standard deviation was crucial for gauging the reliability and consistency of our Hb prediction model. B) MSE quantifies the average squared difference between predicted and actual values in a dataset, measuring the model’s accuracy. A lower MSE indicated predictions closer to the actual data points, while a higher MSE suggested larger prediction errors. C) R-squared (R²) gauges the proportion of variance in the dependent variable explained by the independent variables in the model. It ranges from 0 to 1, with higher values indicating a better fit. D) Regression Plots display measured Hb levels on the x-axis and predicted values on the y-axis. Alignment along the diagonal (x = y) line served as a visual accuracy indicator. Deviations or clusters away from the line indicated performance trends or biases. Additionally, we divided the x-axis into three corridors (Red, Yellow, Green) and denoted decision thresholds to provide a clear, single view understanding of the model’s predictions. *Classification metrics:* A) The confusion matrix evaluates the performance of a machine learning model in multiclass classification tasks, providing a tabular representation of the model’s predictions versus actual class labels. Analyzing these components offers insights into classification accuracy and patterns of misclassification. B) The F1 score combines precision and recall, providing a single performance measure, particularly useful for imbalanced datasets. It ranges from 0 to 1, with higher values indicating better performance. By employing these comprehensive metrics, we ensured a thorough evaluation of the model’s performance, guiding further refinements and optimizations to achieve reliable and accurate Hb level predictions. All models were evaluated using a 10-fold patient-ID–stratified cross-validation scheme. Performance metrics (standard deviation, mean squared error, R², and F1 scores) are reported as the mean values across the validation folds. It should be noted that, during the exploratory development phase of this feasibility study, fold-level metric outputs were not persistently stored; therefore, standard deviations or confidence intervals across folds are not reported. The primary aim of this work was a comparative feasibility assessment under identical evaluation conditions rather than fine-grained statistical benchmarking. Future prospective studies will incorporate systematic logging of fold-level metrics to enable formal uncertainty quantification.

### Comprehensive evaluation of model performance and error analysis

We extensively explored the dataset, considering various parameters in the feature extraction pipeline, model architecture, and model hyperparameters. The results are presented using both qualitative and quantitative methods. Qualitative assessment is done through regression plots, while quantitative evaluation is carried out using validation metrics and confusion matrices. We have considered key quantitative metrics. The R-squared (R2) value of ~0.45 indicates that the model explains 45% of the variance in Hb levels, suggesting a moderate fit. The residual standard deviation reflects an average prediction error of 1.05 mmol/L. These values offer a quantitative basis for assessing the model’s ability to capture the relationship between the input features and Hb levels. We also provided the type 1 and type 2 error rates on each plot to offer a quick quantitative error assessment concerning decision thresholds.

Finally, confusion matrices in our three-way classification (Red, Yellow, Green) provide a comprehensive view of the model’s performance. Each cell in the matrix reveals the number of instances classified as Red, Yellow, or Green, allowing us to assess the model’s prediction characteristics for each class. Given a certain medical context, where misclassifying Yellow as red is less critical than mistaking red for Green, we focus not only on accuracy but also on the trade-offs between type 1 and type 2 errors, especially for the classes that have greater clinical implications. The confusion matrices offer valuable insights into the model’s ability to distinguish between these classes, aiding in the refinement of classification thresholds and strategies to minimize critical misclassifications.

### Post‑hoc QoL–augmented corridor decision layer

The broader concept of HeMonitor extends beyond Hb prediction alone by exploratorily assessing whether non-invasive Hb estimates, when considered alongside contextual patient information, may provide additional value for remote monitoring and clinical reassessment in patients with low Hb levels. To explore the impact of false-positive classifications, in which low Hb estimates may overstate clinical urgency, we introduced an additional post hoc evaluation layer incorporating quantitative assessments of a patient’s quality of life (QoL). This exploratory framework was designed to contextualize model outputs without altering model training, using the following scores: a) The EORTC Global Health Score^8^ is a validated quantitative measure that reflects a patient’s subjective assessment of their overall health and quality of life. It encompasses various aspects of well-being, including physical, psychological, social, and emotional domains. A high Global Health Score indicates a better perceived quality of life, while a lower score suggests a diminished quality of life. b) The EORTC Fatigue Score^8^, on the other hand, focuses specifically on the patient’s level of fatigue and tiredness. It quantifies the extent to which fatigue impacts the patient’s daily life. Fatigue can be a significant concern, especially for individuals with low Hb levels, as it can greatly affect their ability to perform daily activities. A high EORTC Fatigue Score indicates a higher fatigue level. Integrating this metric into the model allows us to assess the severity of fatigue and consider it alongside Hb predictions. The three corridor labels (predicted Hb corridor, Global Health corridor, and Fatigue corridor) are combined using a deterministic, rule-based aggregation system with equal weighting for each input. The final exploratory label is determined by majority vote. In the event of a tie (i.e., one label appears once and the other two are different, resulting in no majority), a conservative tie-breaking rule selects the more severe corridor according to the hierarchy: Red > Yellow > Green. No learned weights or probabilistic modeling are involved in this step; the system is fully transparent and rule-based.

49 patients were randomly selected from the waiting room of the hematological outpatient department at the University Hospital Leipzig. These patients completed the said questionnaires once as part of the study protocol. For patients who underwent transfusion, the EORTC questionnaire was administered before the transfusion to capture their pre-transfusion QoL and fatigue levels. This approach ensures that the QoL data reflects the patient’s condition before potential intervention, enabling a more accurate alignment of QoL scores with Hb levels and transfusion decisions. Exploratory visual analyses were performed in the QoL subset (n = 47) to assess the relationship between Hb levels and EORTC Global Health and Fatigue scores. Formal correlation testing was not pre-specified and is therefore not reported.

## Data Availability

The datasets generated and/or analyzed during the current study are not publicly available due to patient privacy considerations and compliance with the General Data Protection Regulation (GDPR) and institutional ethical requirements. De-identified data may be made available from the corresponding author upon reasonable request for non-commercial research purposes, subject to institutional review and data sharing agreements.

## Code availability

The code developed as part of our Proof of Concept is not currently in a state suitable for direct public release. Our code depends on an internal library that constitutes proprietary intellectual property and, therefore, cannot be made publicly available. As a result, we are unable to provide the code for publication. For specific requests, please contact the authors.
